# Supplementary material for: Global implications of a low soil moisture threshold for microbial hydrogen uptake
Source: Nat Commun. 2025 Dec 12;17:515. doi: 10.1038/s41467-025-67208-3 (PMC12804893; doi:10.1038/s41467-025-67208-3)
Supplement: Supplementary file 1 — Supplementary Information [file 41467_2025_67208_MOESM1_ESM.pdf]

Global Implications of a Low Soil Moisture Threshold for Microbial Hydrogen Uptake

Linta Reji*[1,2,3], Matteo B. Bertagni[1,4,5], Fabien Paulot[6], Qianhui Qin[1,2], Xinning Zhang*[1,2]

[1] High Meadows Environmental Institute, Princeton University, Princeton NJ, USA
[2] Department of Geosciences, Princeton University, Princeton NJ, USA
[3] Current address: Department of the Geophysical Sciences, The University of Chicago, Chicago IL, USA
[4] Department of Civil and Environmental Engineering, Princeton University, Princeton NJ, USA
[5] Current address: Department of Environment, Land and Infrastructure Engineering, Politecnico di Torino, Torino, Italy
[6] Geophysical Fluid Dynamics Laboratory, National Ocean and Atmosphere Administration, Princeton NJ, USA

* Corresponding author

Corresponding author contacts:
Linta Reji (lreji@uchicago.edu), and Xinning Zhang (xinningz@princeton.edu)

Supplementary Information

Supplementary Methods

No diffusive limitation in the incubations within the dry moisture range

Since this work investigates the biotic consumption of H_2, but the overall H_2 uptake from the air results from both diffusive and biotic processes, we demonstrate that, for the small soil volumes and low soil moisture values used in our experiments, the H_2 concentration in the soil air phase (c_{soil}) is essentially equal to that in the headspace volume (c_{air}). This implies that the consumed H_2 is almost immediately replenished from the headspace. To confirm this, we utilize the analytical solution derived by Bertagni et al. (2021), based on the mass conservation of H_2 in soil:

$$c_{soil} / c_{air} = 1 / (1 + v_{BD} (s) / g_s (s))$$

where v_{BD} is the biotic H_2 consumption velocity (representing the rate of H_2 consumption in the soil), and g_s is the diffusive conductance of H_2 in soil. Both v_{BD} and g_s are functions of soil moisture (s). When biotic consumption is significantly faster than diffusion ($v_{BD} \gg g_s$,

i.e., diffusive limitation), then c_{soil} / c_{air} → 0. Conversely, when biotic consumption is slow relative to diffusion, allowing diffusion to replenish soil air H_2, then c_{soil} / c_{air} → 1.

While acknowledging that hydrological soil models are generally applied at the Darcy scale (on the order of cm, due to the continuum hypothesis), we extend their use here to explore the validity of our hypothesis—namely, the limited impact of diffusion—at the smaller scale of our experiments. This exploration, while not aiming for high precision in the strict sense, allows us to assess the relative magnitudes of biotic consumption and diffusion. Using the standard formulation for soil H_2 diffusion as a function of soil moisture (Bertagni et al., 2021), we combined it with two different parameterizations for biotic H_2 consumption: the beta function (Bertagni et al., 2021) and the linear parameterization (Smith-Downey et al., 2006). Applying these parameterizations to the three soils investigated, we obtained the results shown in Figure S1. For meadow and forest soils, we observe minimal diffusive limitation up to 50% saturation. For sand, this threshold occurs around 30% saturation, a value still comfortably within our experimental range. The choice between the beta and linear parameterizations had a negligible effect on the results. These findings provide further confidence in the minimal influence of diffusion in our experiments and strengthen the basis for our biotic consumption results.

Supplementary Figures

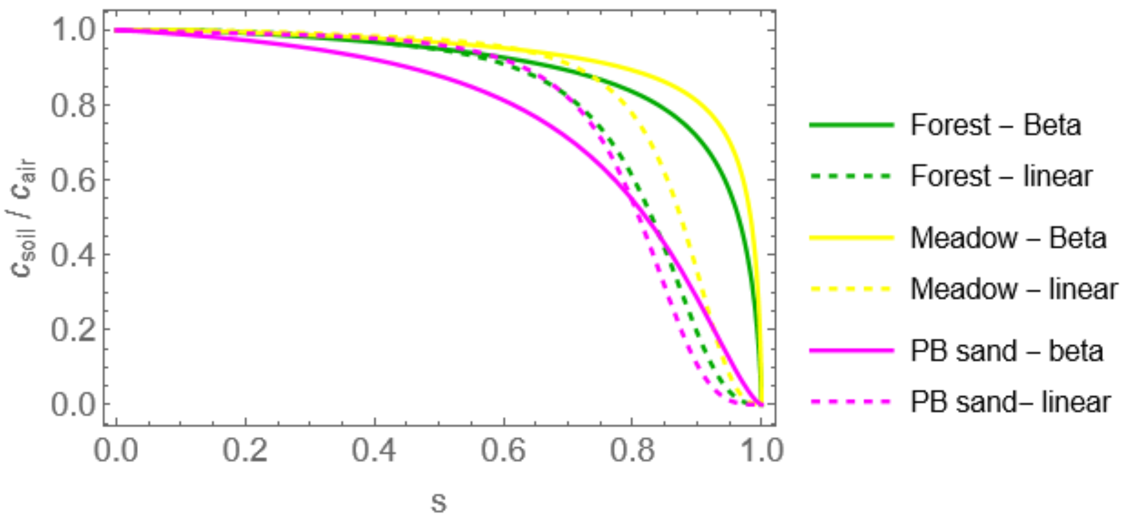

Figure S1: **No diffusive limitation in the experiments within the dry range**. Ratio of H_2 concentrations in soil air to headspace air (c_{soil} /c_{air}) as a function of soil moisture saturation (s) presented as a fraction (1.0 = 100% saturation). See SI Methods for details. Ratios approaching 1.0 indicate minimal diffusive limitation within the experimental setup.

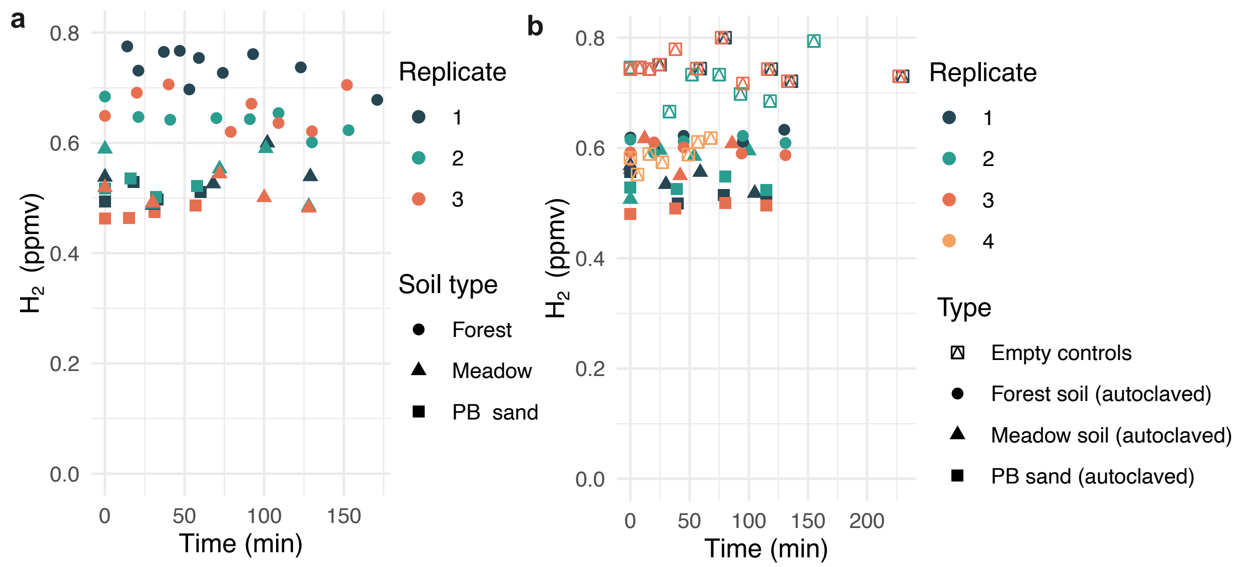

Figure S2: **H_2 oxidation measurements in the control incubations.** Headspace H_2 levels (ppmv) measured in incubations of air-dried soils (a), autoclaved soils (b) and empty bottle controls (b).

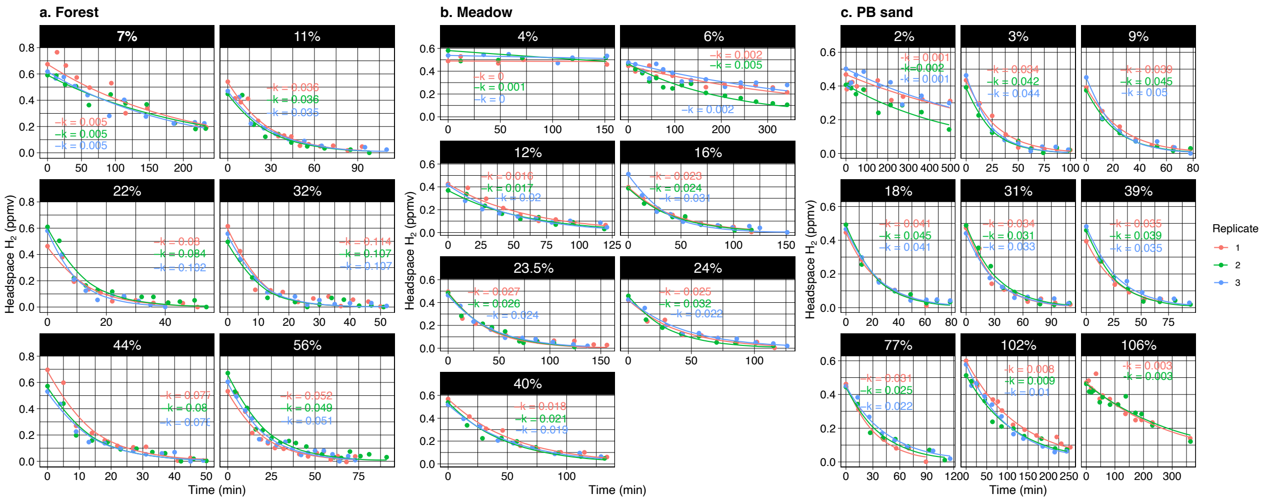

Figure S3: H_2 oxidation curves measured for three replicates each for (**a**) forest, (**b**) meadow, and (**c**) PB sand soils incubated under different moisture levels. The estimated rate constants for each replicate are also indicated on each figure.

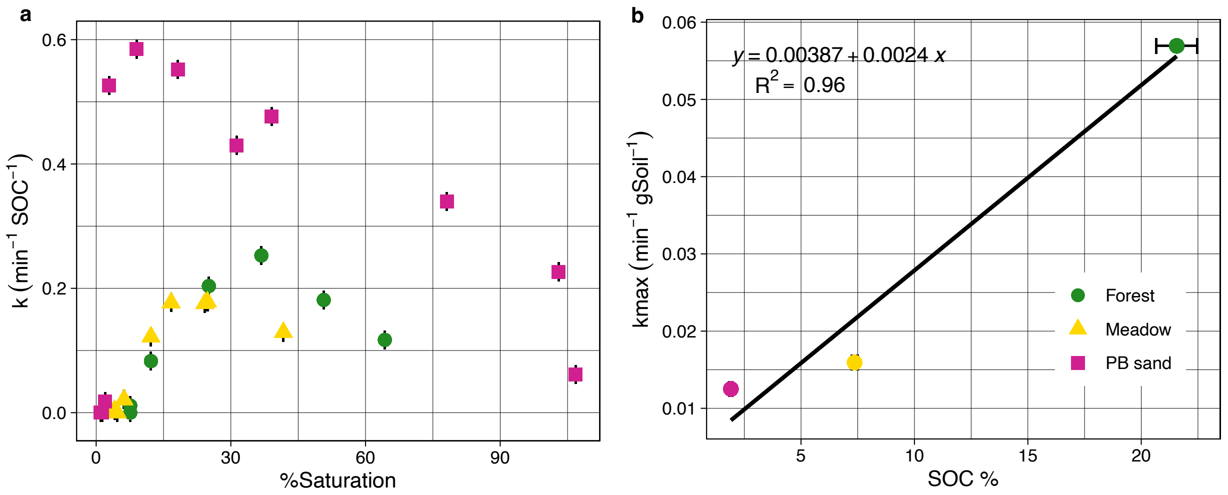

Figure S4: **H_2 oxidation rates increase with SOC**. (a) H_2 oxidation rates across the moisture range scaled by mean estimates of organic matter content (% LOI) for each soil type. Loss-on-ignition (LOI) is calculated as (Pre-ignition weight – Weight after ignition)/Pre-ignition weight). Error bars are standard errors around the mean across three replicates (n=3). (b) A linear fit derived between maximum H_2 oxidation rate (k_{max} min^{-1} gSoil^{-1}) for each soil and SOC (%). In global $v_d(H_2)$ simulations, the linear fit displayed on the plot was used as the SOC modulation.

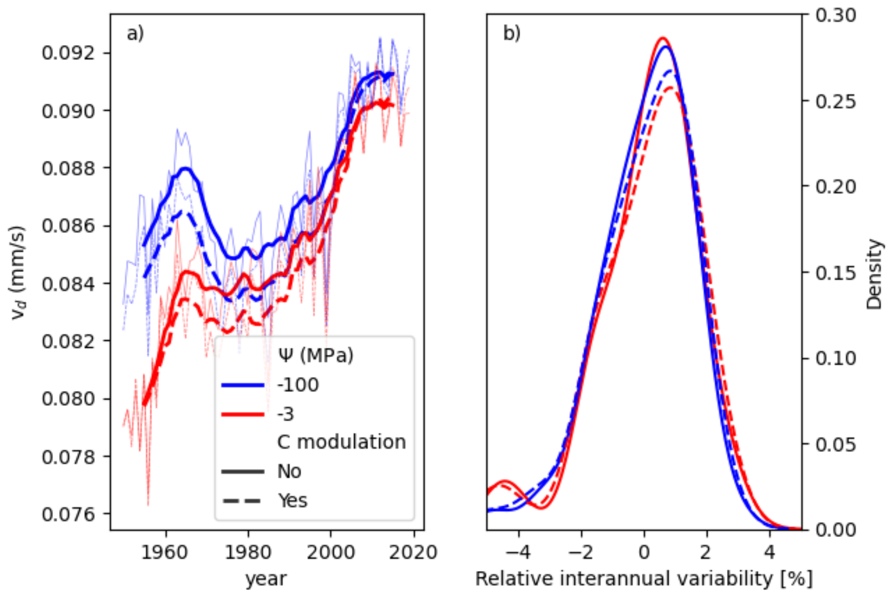

Figure S5: **Effect of the water-stress threshold on vd(H_2) over interdecadal versus interannual timescales**. (a) long-term global timeseries of vd(H_2) for a water stress threshold of -3 MPa (red) vs. -100 MPa (blue). (b) Interannual variability in vd(H_2) relative to the decadal mean. In both panels, dashed and solid lines represent simulations with and without SOC modulation, respectively.

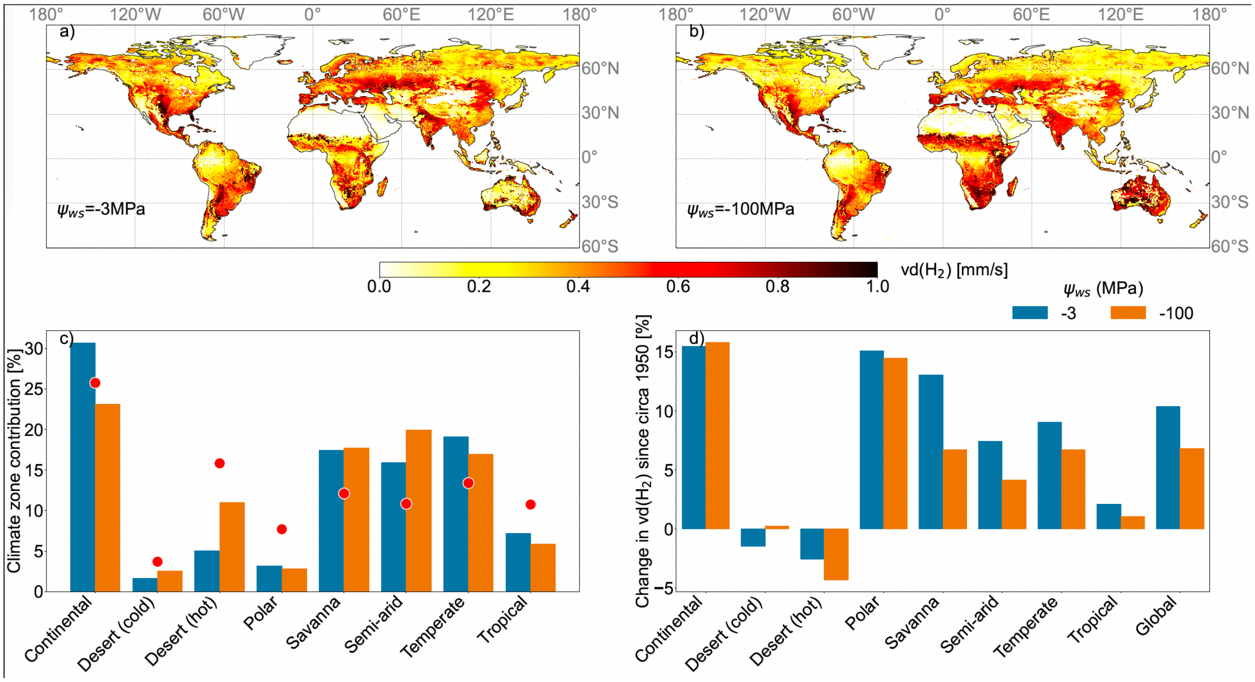

Figure S6: **Simulated deposition velocities ($v_d(H_2)$ mm/s) without a SOC modulation, to compare with Fig. 2**. Spatial distribution of H_2 deposition velocity ($v_d(H_2)$ mm/s) for a water stress threshold of -3 MPa (**a**) vs -100 MPa (**b**) averaged from 2005 to 2019. **c** Fractional contribution of different climate zones to $v_d(H_2)$ for different water stress threshold. Red dots indicate the fractional landmass corresponding to each climate zone north of 60S. **d** Relative change in $v_d(H_2)$ across different climate zones from 1950-1964 to 2005-2019.

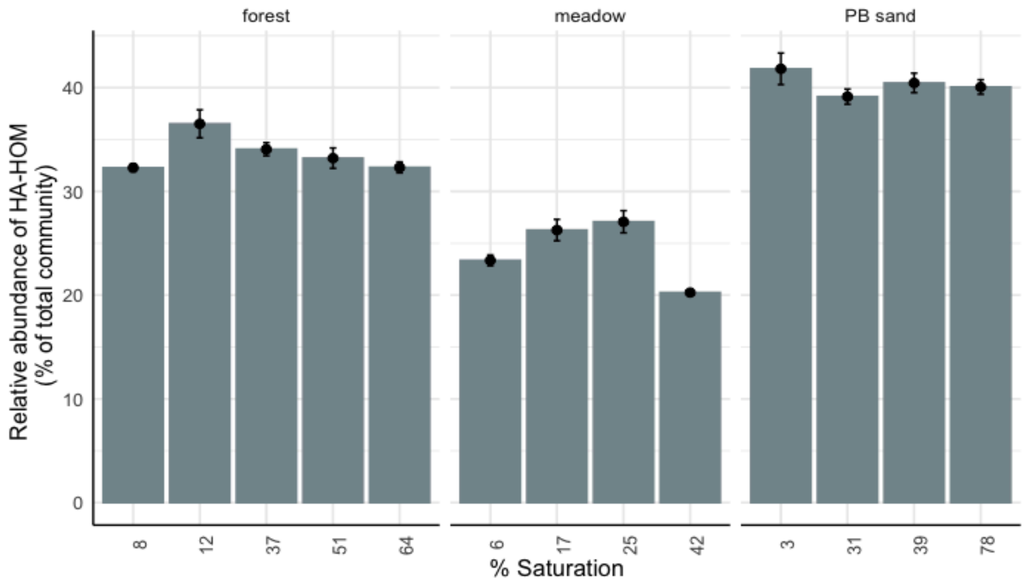

Figure S7: **Relative abundance (% total community) of high-affinity hydrogen oxidizers in each soil type across moisture levels**. We estimated the proportion of HA-HOB by dividing the normalized abundances (RPKM) of Group 1h hydrogenases by the average normalized abundances of the single-copy marker genes *gyrA* and *gyrB*. Error bars represent the propagated standard deviations of the mean *gyrA*-*gyrB* relative abundances, and do not represent variation among biological replicates.

Supplementary Tables

Table S1: Measured water content (as % saturation) and water potential (MPa) of air-dried soils and the moisture-amended incubations corresponding to the moisture activation thresholds for each soil type.

Soil type	Moisture condition	H_2 oxidation observed?	Replicate	Measured %saturation (gravimetric)	Water potential (MPa)
Forest	Moisture added	Yes	1	6.2	-71.5
Forest	Moisture added	Yes	2	6.3	-69.5
Forest	Moisture added	Yes	3	7.3	-68.6
Forest	air-dried	No	1	6.5	-132.4
Forest	air-dried	No	2	6.8	-138.2
Forest	air-dried	No	3	6.6	-140.6
Meadow	Moisture added	Yes	1	5.4	-61.4
Meadow	Moisture added	Yes	2	6.9	-36.1
Meadow	Moisture added	Yes	3	5.7	-53.1
Meadow	air-dried	No	1	4.6	-105.11
Meadow	air-dried	No	2	4.6	-114.44
Meadow	air-dried	No	3	4.4	-113.9
PB_sand	Moisture added	Yes	1	2.4	-89.5
PB_sand	Moisture added	Yes	2	1.9	-72.17
PB_sand	Moisture added	Yes	3	1.7	-89.73
PB_sand	air-dried	No	1	1.4	-118.89
PB_sand	air-dried	No	2	1.4	-120.69
PB_sand	air-dried	No	3	1.2	-121.3

Table S2: H_2 oxidation rates (k, $min^{-1} gSoil^{-1}$), % saturation, and water potential (MPa) measured for the two arid sands collected from Arizona. Limits of detection for H_2 oxidation rates: around 0.00004 ppm min^{-1} g-$Soil^{-1}$ for AZ soil 1, and 0.00005 ppm min^{-1} g-$Soil^{-1}$ for AZ soil 2.

Soil sample	Moisture condition	H_2 oxidation measured?	Replicate	Measured %saturation	Water potential (MPa)	Measured rate, k ($min^{-1} gSoil^{-1}$)
AZ soil 1	Moisture added	No	1	8.24	-128.45	0
AZ soil 1	Moisture added	No	2	7.81	-139.47	0
AZ soil 1	Moisture added	No	3	9.66	-104.17	0
AZ soil 1	Moisture added	No	1	8.91	-124.6	0
AZ soil 1	Moisture added	No	2	10.34	-87.8	0
AZ soil 1	Moisture added	No	3	9.50	-98.73	0
**AZ soil 1**	**Moisture added**	**Yes**	**1**	12.22	**-44.31**	**0.00089**
**AZ soil 1**	**Moisture added**	**Yes**	**2**	12.36	**-32.57**	**0.00115**
**AZ soil 1**	**Moisture added**	**Yes**	**3**	13.07	**-22.08**	**0.00143**
AZ soil 2	Moisture added	No	1	11.20	-90.42	0
AZ soil 2	Moisture added	No	2	9.11	-136	0
AZ soil 2	Moisture added	No	3	9.61	-108.89	0
AZ soil 2	Moisture added	No	1	9.91	-108.8	0
AZ soil 2	Moisture added	No	2	12.46	-61.23	0
**AZ soil 2**	**Moisture added**	**Yes**	**3**	11.23	**-75.69**	**0.0012**
**AZ soil 2**	**Moisture added**	**Yes**	**1**	12.68	**-58.32**	**0.00009**
**AZ soil 2**	**Moisture added**	**Yes**	**2**	13.13	**-49.12**	**0.00007**
**AZ soil 2**	**Moisture added**	**Yes**	**3**	13.46	**-36.52**	**0.00023**

Table S3: Summary of DNA and RNA extractions. Soil samples from triplicate moisture incubations were pooled for extraction for the meadow soil and PB sand, since individual replicates did not have sufficient yield (e.g., PB sand 1% incubations). Samples chosen for metagenomes and metatranscriptomes are highlighted in green.

Soil	Average saturation	Replicates pooled for extraction?	Amount of soil extracted from (g)	DNA yield (ng/ul)	RNA yield (ng/ul)
PB sand	~1%	No	0.63	6.4	unsuccessful
			0.54	-0.3	unsuccessful
			0.53	1.8	unsuccessful
PB sand	~9%	No	0.54	1.6	unsuccessful
			0.52	2.8	unsuccessful
			0.43	3.4	unsuccessful
PB sand	~3%	Yes	1.41	17.6	7.2
PB sand	~18%	Yes	1.27	20.3	2.7
PB sand	~31%	Yes	1.24	15.3	8.6
PB sand	~39%	Yes	1.3	28.7	12.7
PB sand	~102%	Yes	1.06	14	1.8
PB sand	~78%	Yes	1.22	18.1	12.1
Meadow	~6%	Yes	1.24	171.1	72.8
Meadow	~12%	Yes	1.23	306.5	47.5
Meadow	~16%	Yes	1.26	265.5	76.9
Meadow	~24%	Yes	1.26	366.9	103.7
Meadow	~24%	Yes	1.21	163	98.4
Meadow	~40%	Yes	1.22	232.9	99
Forest	~7%	No	0.32	90.1	21.4
			0.37	148.1	25.3
			0.27	86.6	46.5
Forest	~11%	No	0.31	65.7	19.5
			0.31	147.5	50
			0.28	45.2	13.2
Forest	~22%	No	0.31	82	27.2
			0.29	48.2	12.8
			0.33	79.8	24.7
Forest	~32%	No	0.32	94.9	15.4
			0.28	24.5	26.5
			0.26	11.1	31.7
Forest	~44%	No	0.31	83.4	28.7
			0.33	58.5	25.5
			0.37	119.2	37.8
Forest	~56%		0.29	81.6	19.6

Table S4: Summary of metagenome assembly and binning results.

Soil type	Metagenome ID	Number of reads	Number of contigs	N50	Group 1h homologs	Group 2a homologs	Number of medium -to-high quality MAGs	Number of HA-HOB MAGs
Meadow	M_7	145915202	790560	1637	139	0	4	2
	M_20	148173626						
	M_30	136510560						
	M_50	148907052						
PB sand	PB_5	169038130	1173301	2587	297	3	14	5
	PB_30	193315878						
	PB_40	130919336						
	PB_70	142585812						
Forest	F_6	165002440	1860510	2087	185	0	18	5
	F_10	124949482						
	FS_30	238887430						
	F_40	143549064						
	F_orig_30	172195028						
